# Korean Red Ginseng Improves Oxidative Stress-Induced Hepatic Insulin Resistance via Enhancing Mitophagy

**DOI:** 10.3390/foods13132137

**Published:** 2024-07-05

**Authors:** Nodir Rustamov, Yuanqiang Ma, Jeong-Su Park, Feng Wang, Hwan Ma, Guoyan Sui, Gahye Moon, Hwan-Soo Yoo, Yoon-Seok Roh

**Affiliations:** College of Pharmacy and Medical Research Center, Chungbuk National University, Cheongju 28160, Republic of Korea; nodirrustamov.nr@gmail.com (N.R.); yuanqiangma123@gmail.com (Y.M.)

**Keywords:** insulin resistance, mitophagy, *Korean Red Ginseng*, PINK1/Parkin pathway, oxidative stress

## Abstract

This study explored the potential of saponins from Korean Red Ginseng to target the PINK1/Parkin mitophagy pathway, aiming to enhance insulin sensitivity in hepatocytes—a key factor in metabolic disorders like metabolic dysfunction-associated steatotic liver disease (MASLD) and type 2 diabetes. Results from both in vitro and in vivo experiments showed increased expression of PINK1 and Parkin, activating mitophagy and reducing oxidative stress through reduction in mitochondrial and total reactive oxygen species. Additionally, improvements in insulin signaling were observed, including the upregulation of phosphorylated IRS and AKT, and downregulation of gluconeogenic enzymes, underscoring the saponins’ efficacy in boosting insulin sensitivity. The findings highlighted Korean Red Ginseng-derived saponins as potential treatments for insulin resistance and related metabolic conditions.

## 1. Introduction

Insulin resistance is a condition where the body’s cells become less responsive to insulin, leading to elevated blood sugar levels and disrupted glucose and lipid metabolism [1]. This state not only precedes type 2 diabetes but also plays a significant role in the pathogenesis of various metabolic diseases [2]. In MASLD, insulin resistance contributes to lipid accumulation in the liver, causing inflammation and liver damage [3]. It is a key factor in the development of type 2 diabetes through its role in chronic hyperglycemia and acts as a risk factor for cardiovascular disease by promoting dyslipidemia, hypertension, and endothelial dysfunction, which can lead to atherosclerosis and heart complications [4,5]. Impaired insulin action affects glucose uptake and metabolism, underlining its pivotal role in metabolic health and disease progression.

This complex interplay between insulin resistance and metabolic diseases highlights the importance of understanding underlying mechanisms, including the role of mitochondrial health [6,7,8]. Mitochondria, the cell’s powerhouse, are central to energy production and metabolic regulation. Their dysfunction, marked by reduced ATP production and increased oxidative stress, is intricately linked to insulin resistance [9]. Mitochondrial oxidative stress, characterized by an overproduction of reactive oxygen species (ROS), contributes to cellular damage and impaired insulin signaling. Normally, mitochondria generate ROS as byproducts of oxidative phosphorylation [10]. Under physiological conditions, these ROS are neutralized by antioxidant defense systems. However, under pathological conditions, such as high-fat diets, excessive glucose, and inflammation, mitochondrial electron transport chain (ETC) becomes overactive or dysfunctional, leading to excessive ROS production [11,12]. This imbalance overwhelms the cell’s antioxidant defenses, causing oxidative damage to mitochondrial DNA, proteins, and lipids [13]. This oxidative stress not only disrupts mitochondrial function but also exacerbates inflammation and metabolic dysregulation, further promoting insulin resistance [14]. In this context, maintaining mitochondrial function emerges as a crucial strategy for mitigating insulin resistance and its associated metabolic disorders. Although the significance of mitochondrial dynamics in metabolic health is widely accepted, the exact mechanisms by which mitochondrial health influences insulin resistance and how mitophagy plays into this equation remain areas of active investigation, underscoring the complexity of metabolic regulation and the potential for targeted therapeutic strategies.

By exploring cellular mechanisms and their implications for metabolic health, traditional medicines offer a rich avenue for discovery. Specifically, Korean Red Ginseng (*Panax ginseng Meyer*) has been recognized for its health-promoting compounds [15,16,17]. Among their constituents—saponins, *Ginsenosides*, stand out for their potential properties, particularly in terms of metabolic health [16,18,19]. Saponin uptake and glycosides are known for their surfactant properties, enabling them to modulate biological activities. Ginsenosides have several beneficial effects, including improving insulin sensitivity, enhancing glucose uptake, and protecting against oxidative stress by reducing reactive oxygen species [20,21,22]. Additionally, they possess anti-inflammatory properties that help mitigate the chronic inflammation associated with metabolic disorders [23]. Ginsenosides also contribute to improved lipid metabolism by reducing triglyceride and cholesterol levels, enhancing mitochondrial function, and supporting cardiovascular health by improving endothelial function and reducing hypertension [18,24,25]. While the broader health benefits of ginseng-derived saponins are well documented, ongoing research aims to pinpoint the precise molecular pathways through which they operate, particularly in relation to mitophagy. These studies seek to elucidate how ginsenosides modulate mitochondrial function, reduce oxidative stress, and promote the removal of damaged mitochondria, thereby improving insulin sensitivity and metabolic health.

## 2. Materials and Methods

### 2.1. Preparation of Red Korean Ginseng and Saponin Extract

In the Korea Ginseng Corporation’s Korean Red Ginseng manufacturing facility located in Buyeo, Chungnam, Korea, fresh ginseng roots underwent a series of processing steps to produce Korean Red Ginseng. Initially, these roots were cleaned and steamed over a 4 h period, with the temperature gradually increasing from 50 °C to 98 °C. Following this, the roots were subjected to an initial drying phase at a temperature range of 60–70 °C for 15 h. A subsequent drying phase took place in a sealed chamber where the roots remained at 50 °C for a duration of 5 days. This process resulted in the formation of Korean Red Ginseng (RG) roots.

To obtain the Korean red ginseng extract, the RG was methodically extracted with distilled water seven times, each time at 87 °C, over a 12 h span. The collected extracts were then combined, filtered, and concentrated. The RG extract was then utilized to segregate the non-saponin and saponin components through an adsorption chromatography method. This involved the use of Dion HP20 resin, produced by Mitsubishi Chemical Industries in New York, NY, USA. The extract was first diluted to a 10% concentration with water and filtered. This diluted mixture was passed through the HP20 resin for adsorption. The elution process involved water, a 30% ethanol-water solution, and a 95% ethanol-water solution, in that order. The combined fractions from the water and 30% ethanol elutions were concentrated and underwent spray-drying, yielding the non-saponin fraction, termed NS-RG. Conversely, the 95% ethanol fraction, post-concentration, and spray-drying resulted in the saponin fraction, labeled S-RG. Quantitative analysis of ginsenoside and red ginseng polysaccharides in non-saponin and saponin fractions obtained from High-Performance Liquid Chromatography (HPLC) was provided in Supplementary Material.

### 2.2. Cell Culture

Human hepatocyte cell lines, Hep3B and HepG2, were cultured in Dulbecco’s Modified Eagle’s Medium (DMEM) supplemented with 10% fetal bovine serum (FBS) in a 5% CO_2_ atmosphere at 37 °C. To study the mitophagy-inducing effect of saponin extract, Hep3B cells were transfected with pLVX-puro-mtKeima, then incubated with carbonyl cyanide m-chlorophenylhydrazone (CCCP, 3 uM) and two concentrations of Saponin extract 20 ug/mL (RG low) or 40 ug/mL (RG high) for 24 h. To detect the effect of Saponin extract on mtROS production, Hep3B cells were treated with Palmitic Acid (PA, 300 uM) and Saponin extract for 16 h. To detect the effect of Saponin extract on total ROS level, Hep3B cells were treated with PA and saponin extract for 16 h. To detect the effect of Saponin extract on insulin resistance, Hep3B cells were treated with Saponin extract for 24 h and were incubated with 10 nm Insulin before collecting.

### 2.3. Animal Experiment and Ethical Approval

8-week-old male C57BL/6 mice were acquired from Samtako Bio Korea in Osan, Korea. The Chungbuk National University Institutional Animal Care and Use Committee (IACUC) granted approval for all animal-related experiments. The IACUC of the Laboratory Animal Research Center at Chungbuk National University in Cheongju, Korea, also sanctioned the protocol (Ethical approval No. CBNUA-1203-18-02). The mice were kept under a 12 h light/dark cycle at 21 ± 2 °C in a specific-pathogen-free environment. To -avoid contamination, the experimental room maintained high pressure. For the study, mice were grouped into three categories, each consisting of 2 mice per group: (1) Mice were given water; (2) Mice were given a low dose of saponin extract (50 mg/kg); (3) Mice were given a high dose of saponin extract (150 mg/kg). For the subsequent 4 weeks, they received either water or specific doses of saponin extract (50 or 150 mg/kg) daily via oral gavage.

### 2.4. Chemicals

Dulbecco’s Modified Eagle’s Medium (DMEM), Corning^®^, Glendale, CA, USA; Fetal Bovine Serum (FBS), Corning^®^, Glendale, CA, USA; M199 medium, Corning^®^, Glendale, CA, USA; Penicillin-Streptomycin solution 100× (PS), Corning^®^, Glendale, CA, USA; TB Green Premix Ex Taq II, TAKARA, Shiga, Japan; Carbonyl Cyanide m-Chlorophenylhydrazone (CCCP), Sigma-Aldrich, St. Louis, MO, USA; Metformin hydrochloride Sigma-Aldrich, St. Louis, MO, USA; MitoSOXTM Invitrogen by Thermo Scientific, Waltham, MA, USA; Palmitic Acid, Sigma-Aldrich, St. Louis, MO, USA; Lipofectamine™ 3000 Transfection Reagent Invitrogen by Thermo Scientific, Waltham, MA, USA; Primer ScriptTM RT Reagent Kit with gDNA Eraser, Shiga, Japan; BCA Protein Assay Kit, Thermo Scientific, Waltham, MA, USA; Anti-Pink1 Abclonal (A7131); Anti-Parkin Abcam (AB15494); Actin Santa Cruz (A5441); TOM40 Santa Cruz (sc-365467); TIM23 Abclonal (A8688); P62 Cell Signaling Technology, Danvers, MA, USA; LC3B Cell Signaling Technology (#3868); Tom20 Santa Cruz (sc-17764); Tom70 Santa Cruz (sc-390545); P-IRS Abcam (Ab5599); IRS Abcam (Ab131487); P-JNK Abclonal (AP0276); JNK Abclonal (A4867); p-AKT Cell Signaling Technology (4060S); AKT Cell Signaling Technology (9272S), p-AMPK Abcam (40H9), AMPK Cell Signaling Technology (4060S).

### 2.5. Western Blot Analysis

Western blotting was performed to evaluate the corresponding proteins. Proteins were extracted from cells or tissues and lysed in Radioimmunoprecipitation assay buffer (RIPA) containing protease and phosphatase inhibitors. Lysis was conducted on ice for a duration of 30 min. Following lysis, the samples were subjected to centrifugation at 13,000 rpm for 15 min at 4 °C to collect the protein-rich supernatant. The protein concentration of the supernatant was determined using a BCA Protein Assay Kit (Thermo Fisher Scientific Inc., Waltham, CA, USA). Thereafter, equivalent concentrations of protein samples from different groups were prepared for electrophoresis and then imprinted onto a membrane. Next, the membranes underwent a blocking step in a non-fat milk powder solution at approximately 25 °C for over 2 h or in a 5% Bovine Serum Albumin (BSA) solution at 4 °C overnight. The membranes were then probed with primary antibodies diluted in 5% BSA in tris-buffered saline/Tween (TBST), followed by secondary antibodies diluted in 2% BSA overnight at 4 °C or 2% skimmed non-fat milk in TBST for 2 h. All antibodies were diluted at a ratio according to manufacturer guidance. To remove unbound antibodies, membranes were washed three times for 10 min each with TBST.

### 2.6. Quantitative Real-Time Polymerase Chain Reaction (PCR) Analysis

Cells and liver tissues were homogenized in RiBoEx (Geneall Biotechnology Co., Ltd., Seoul, Republic of Korea; Cat. No. 301-001). Cell lysates were combined with 200 uL chloroform and then centrifuged at 12,000× *g* for 15 min at 4 °C. After this, total RNA was isolated through phase separation, binding, washing, and elution using the Hybrid-R™ kit (Geneall Biotechnology Co. Ltd., Seoul, Republic of Korea) according to the manufacturer’s guidance. This RNA was then reverse transcribed to complementary DNA (cDNA) using the PrimerScriptTM RT Reagent Kit with Genomic DNA (gDNA) Eraser (TAKARA Bio Inc. Shiga, Japan; Cat. No. RR037A). RT-qPCR was executed using the CFX Connect Real-Time PCR Detection System (Bio-Rad, Hercules, CA, USA). The relative expression of targeted genes was standardized to the glyceraldehyde 3-phosphate dehydrogenase (GAPDH, internal control) expression. The specific primer sequences are Hm GAPDH R: GAAGATGGTGATGGGATTTC, F: GAAGGTGAAGGTCGGAGTC. Ms GAPDH R: TTGCTGTTGAAGTCGCAGGAG, F: TGTGTCCGTCGTGGATCTGA. Ms PEPCK R: ATGACACCCTCCTCCTGCAT, F: CAGGAAGTGAGGAAGTTTGTGG, Ms G6Pase R: TGCAGCTCTTGCGGTACATG, F: TGCAGCTCTTGCGGTACATG, Hm G6Pase R: GAGCCACTTGCTGAGTTTCC, F: GTCCACATTGACACCACACC, Hm PEPCK R: TGGTCTCAGCCACATTGGTA, F: AACCCTGAGAACGGCTTCTT.

### 2.7. Flow Cytometry (FACS) Detection of Mitophagy in Hepatocytes

MT-Keima Hepatocytes were incubated with 0.25% trypsin for a duration of 3 min to allow for enzymatic detachment. In the case of primary MT-Keima hepatocytes, they were treated with a lower concentration of 0.05% trypsin and incubated for a shorter duration of 1 min. Following the trypsin treatment, cells were centrifuged at 7000 rpm for 3 min, resulting in a consolidated cell pellet. The pellet was subsequently resuspended in a FACS buffer composed of 0.5% Bovine Serum Albumin (BSA) and 0.2% EDTA, to achieve a homogenous mixture. Mitophagy within the MT-Keima Hepatocytes was then assessed using the guava easyCyte™ Flow Cytometry System (Millipore Corporation, Billerica, MA, USA).

### 2.8. Detection of Mitochondrial and Total ROS

Hep3B cells were treated either with 300 µM PA alone or co-treated with Saponin at concentrations of 20 µg/mL or 40 µg/mL for a duration of 16 h. Following treatment, for FACS cells were harvested using trypsin and collected into microtubes. For the assessment of mitochondrial reactive oxygen species (mtROS) production and Total ROS, cells were stained with MitoSOX and H2DCFDA, respectively, and incubated at 37 °C for 20 min. Subsequent mtROS detection was carried out using Leica DFC7000T and/or the Guava easyCyte™ Flow Cytometry tool, Merck KGaA, Darmstadt, Germany.

### 2.9. pMitotimer Transfection in Hep3B Cells

Hep3B cells were transfected with the pMitotimer plasmid using the Lipofectamine 3000 transfection kit and incubated for 18 h. Post-transfection, cells underwent selection by treatment with Geneticin™ for the subsequent 24 h. Following selection, cells were either treated with metformin alone or in combination with Saponin at concentrations of 20 µg/mL or 40 µg/mL. The expression of pMitotimer was visualized using a Laser Microscope from Leica DFC7000T, Leica Microsystems GmbH, Wetzlar, Germany.

## 3. Results

### 3.1. Red Korean Ginseng Saponin Enhances Hepatic Mitophagy

**Figure 1:**
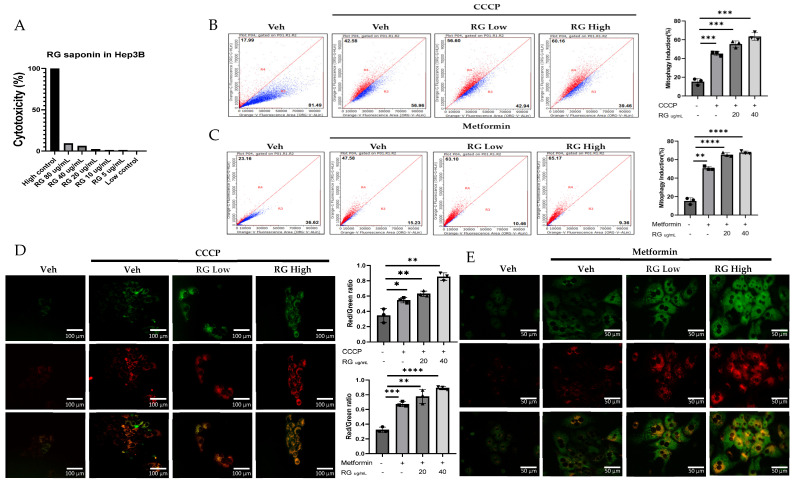

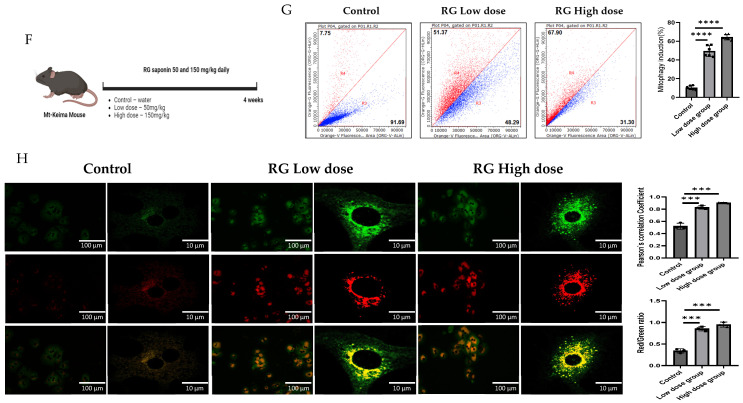
RG Ginsenosides induce mitophagy in cells and mice. (**A**) Cytotoxicity assay was conducted to assess the effects of various concentrations of RG saponin. (**B**,**C**) Flow cytometry analysis revealed the results of RG saponin treatment on the MT-Keima system in Hep3B cells and primary hepatocytes. (**D**,**E**) Microscopic imaging was utilized to observe RG saponin-induced mitophagy in Mt-Keima Hep3B cells and hepatocytes. (**F**) A diagram outlined the treatment model (50 mg/kg and 150 mg/kg) and duration for Mt-Keima mice, detailing the regimen. (**G**) Flow cytometry data were collected from hepatocytes derived from mice post-gavage with RG saponin. (**H**) Microscopic imaging captured the effects on hepatocytes from mice after gavage. The data are presented as means ± SEM, with significance levels marked as * *p* < 0.05, ** *p* < 0.01, *** *p* < 0.001 and **** *p* < 0.0001.

Investigations into the effects of ginsenosides from Red Korean Ginseng on mitophagy involved cellular and in vivo studies using Mt-Keima Hep3B cells and primary hepatocytes treated with 20 µg/mL (RG Low) and 40 µg/mL (RG High) ginsenoside concentrations, which were selected based on cytotoxicity assays conducted on hepatocytes to ensure effective yet non-toxic doses. (Figure 1A). Results, depicted in Figure 1B–E, showed a clear dose-dependent increase in mitophagy across both cell types, demonstrating ginsenosides’ significant role in enhancing mitophagy activity.

Transitioning to a more holistic, in vivo study, Mt-Keima mice were chosen as the model organisms. These mice were administered ginsenosides at two varied dosages: a moderate 50 mg/kg (RG Low dose) and a more substantial 150 mg/kg (RG High dose), spanning across a diligent 4-week regimen. These dosages were determined based on in vivo studies that utilized ginsenosides/extracts and our previous research on the effect of Ginseng on NAFLD. In these studies, similar doses demonstrated a significant preventative effect without adverse impacts on mice viability [26,27,28,29,30]. Post-administration, hepatocytes harvested from these mice unveiled significant findings. Detailed in Figure 1F–H, the hepatocytes from the higher dosage group demonstrated a more potent mitophagy reaction, echoing the in vitro revelations. This consistent trend across both in vitro and in vivo studies underscores the pivotal role ginsenosides play in modulating mitophagy.

### 3.2. Korean Red Ginseng Induces PINK1/Parkin-Dependent Hepatic Mitophagy

**Figure 2:**
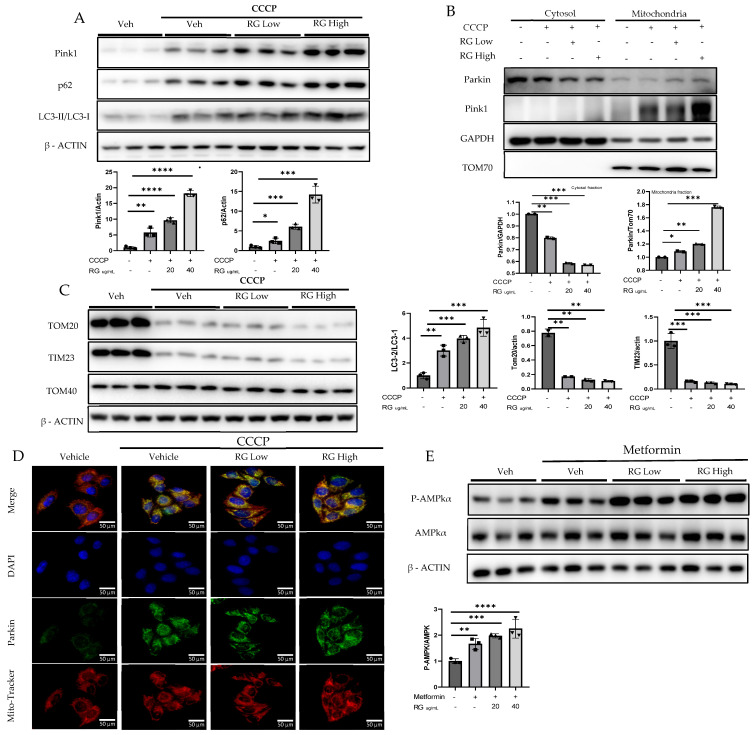
RG Ginsenosides promote mitophagy through Parkin-dependent pathway. (**A**,**B**) Post-treatment representative Western blotting analysis of mitophagy markers PINK1, Parkin, p62, and LC3 I-II in both whole cell lysate and the mitochondrial fraction. (**C**) Western blotting analysis was utilized to check post-treatment expression of mitochondrial complex proteins TOM20, TOM40, and TIM23. (**D**) Immunofluorescence visualization of Parkin accumulation. (**E**) Western results in response to RG saponin treatment P-AMPK, AMPK. The data are expressed as means ± sem. * *p* < 0.05, ** *p* < 0.01, *** *p* < 0.001 and **** *p* < 0.0001.

To discern the impact of RG saponins on hepatic cellular function, Hepatocyte cells were treated with two concentrations of RG saponins. Upon treatment with RG saponin, a significant escalation in the accumulation of Pink1 was noted in both the total lysate and mitochondrial fraction (Figure 2A,B). Parallel to this increase in Pink1 levels, there was an enhanced accumulation of Parkin on the mitochondrial surface. This was corroborated at the protein expression level, as evidenced by the immunofluorescence results (Figure 2B,E). The simultaneous upsurge of Pink1 and Parkin suggests a potential activation of the mitophagy pathway in Hepatocyte cells following RG saponin administration.

Supporting the hypothesis of augmented mitophagy, there was an evident increase in autophagosome formation. A pronounced formation of autophagosomes was identified, which increased the autophagic activity. Delving deeper into autophagy markers, we found a significant increase in the expression of P62. Moreover, the LC3-II/LC3-I ratio showed a spike, further corroborating our hypothesis. Alongside these findings, a decline in specific mitochondrial protein complexes, particularly TOM20 and TIM23, was observed (Figure 2C). Such decreases typically suggest processes such as mitochondrial fragmentation or even degradation, implying that mitochondria are potentially being targeted for removal.

Following RG saponin treatment, significant changes in cellular energy regulation were observed, particularly in the upregulated expression of P-AMPKα (Figure 2D). The study indicates that treatment with RG saponin enhances the cellular process of mitophagy, leading to increased removal of damaged mitochondria and changes in energy regulation within Hepatocytes.

.

### 3.3. Impact of RG Saponin on Mitochondrial Health and Oxidative Stress Regulation in Hepatic Cells

**Figure 3:**
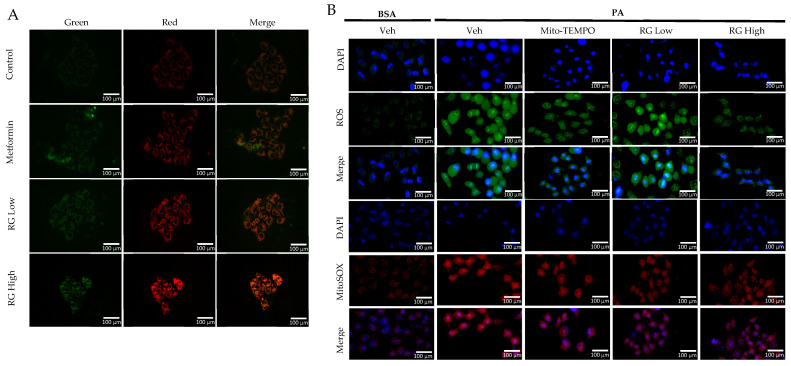

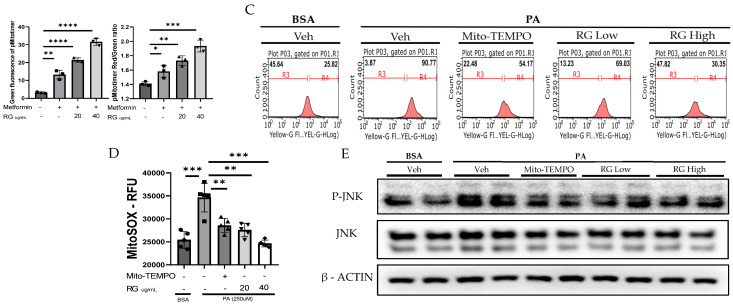
RG Ginsenosides decrease production of mtROS and tROS. (**A**) Microscopic imaging showcased mitochondrial biogenesis in Hep3B cells transfected with the pMitotimer plasmid. (**B**) mtROS and total ROS levels were monitored in Hep3B cells after 16 h of treatment with palmitic acid (PA) alone or in co-treatment with RG saponin. (**C**) Flow cytometry was utilized to detect mtROS production in Hep3B cells following treatment with PA and RG saponin. (**D**) Colorimetric analysis of MitoSOX-stained Hep3B cells was conducted to quantify mtROS levels. (**E**) Western blot analysis was performed to evaluate the protein expression of P-JNK as an oxidative stress marker after co-treatment with PA and RG saponin. The data are expressed as means ± SEM, with significance denoted by * *p* < 0.05, ** *p* < 0.01, *** *p* < 0.001 and **** *p* < 0.0001.

Utilizing the pMitotimer tool in RG saponin-treated Hep3B cells [31], our observations indicated enhanced mitochondrial turnover, suggesting a rejuvenation process where older or damaged mitochondria are replaced by healthier ones (Figure 3A) [32]. This points to RG saponin’s significant role in promoting mitochondrial health and effectively managing mtROS levels, which are vital in preventing metabolic complications and insulin resistance associated with excessive mtROS [33].

Further, our use of MitoSOX staining and flow cytometry to measure mtROS in Hep3B cells treated with RG saponin revealed a pronounced reduction in mtROS, emphasizing RG saponin’s capacity to bolster mitochondrial health (Figure 3B). This reduction is particularly important as unchecked mtROS can lead to total cellular ROS build-up, intensifying oxidative stress. This aspect was corroborated by DCFDA staining (Figure 3B–D), showing a dose-dependent decrease in total cellular ROS following RG saponin treatment. Complementing these findings, our exploration of oxidative stress biomarkers showed a significant decrease in P-JNK expression [34], an indicator of oxidative stress upregulated in response to reactive species, after RG saponin treatment (Figure 3E). Together, these results underscore RG saponin’s potential for mitigating oxidative damage and enhancing cellular health by improving mitochondrial function and reducing oxidative stress.

### 3.4. Korean Red Ginseng Improves Hepatic Insulin Signaling and Inhibits Gluconeogenesis

**Figure 4:**
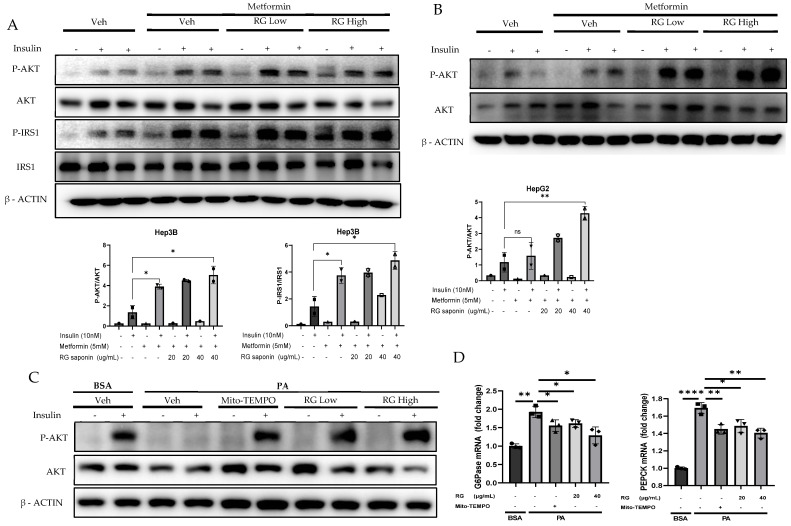
RG Ginsenosides improves Insulin sensitivity and attenuate Gluconeogenesis. (**A**,**B**) Western blotting analysis of insulin sensitivity markers P-IRS, IRS, P-AKT, AKT in Hep3B and HepG2 cell lines. Cells were treated with RG saponin for 24 h and were stimulated with 10 nM Insulin for 15 min before collection. (**C**) Western blotting demonstrated P-AKT expression in Primary Hepatocytes after treatment with PA and RG saponin. (**D**) mRNA expression analysis of genes involved in gluconeogenesis, G6Pase, PEPCK. The data are expressed as means ± sem. * *p* < 0.05, ** *p* < 0.01, **** *p* < 0.0001.

Building on previous findings that highlighted the downregulation of P-JNK by RG saponin treatment, our investigation further explored its impact on insulin signaling pathways in hepatocytes. Prior to insulin stimulation, hepatocytes treated with RG saponin demonstrated a significant increase in the phosphorylation levels of insulin receptor substrate (P-IRS) and protein kinase B (P-AKT) in a dose-dependent manner (Figure 4A–C). This upregulation, following the reduction of P-JNK, amplifies insulin signaling pathways, reinforcing RG saponin’s role in enhancing cellular insulin sensitivity [35]. These observations suggest RG saponin’s potential to improve insulin signal transduction within hepatocytes, offering promising insights into its capacity to modulate key regulatory mechanisms of insulin sensitivity.

These findings highlight RG saponin’s comprehensive role in enhancing insulin signaling and metabolic balance. By boosting P-IRS and P-AKT levels and reducing key gluconeogenic enzymes through the AKT-FOXO1 pathway [36,37], alongside glycolysis modulation, RG saponin demonstrates its efficacy in improving insulin sensitivity. The observed decrease in gluconeogenic enzyme mRNA, like G6Pase and PEPCK (Figure 4D), showcases RG saponin’s ability to influence insulin pathways and manage metabolic disorders by fine-tuning critical metabolic processes.

## 4. Discussion

In this study, we explored the multifaceted role of RG saponin, derived from *Korean Red Ginseng*, in modulating mitochondrial dynamics, oxidative stress, and insulin signaling pathways, which are pivotal in the pathogenesis of insulin resistance and metabolic disorders.

Our study highlighted the effectiveness of ginsenosides from RG in enhancing hepatic mitophagy, a vital process for maintaining mitochondrial integrity and function. We observed a dose-dependent increase in mitophagy both in vitro and in vivo, emphasizing the significant role ginsenosides play in this cellular pathway. This aligns with previous research, underscoring the critical nature of mitochondrial quality control in cellular metabolism and the ability of natural compounds to influence these mechanisms. Further investigation into RG saponin’s molecular effects revealed a dose-dependent increase in PINK1 and Parkin accumulation in hepatocytes, key indicators of mitophagy activation [38]. This is noteworthy given the essential role of the PINK1/Parkin pathway in mitophagy and its relevance to metabolic health.

Additionally, the upregulation of autophagy markers, such as P62 and the LC3-II/LC3-I ratio, coupled with a reduction in mitochondrial protein complexes, supports the notion that RG saponin promotes mitophagy, thereby improving mitochondrial quality and function, and highlights its preventative potential in managing metabolic disorders through the modulation of mitochondrial dynamics.

The choice of 8-week-mice corresponds to young adulthood in humans (<20 years) and is optimal for metabolic studies due to stable physiological systems and minimal age-related variability. Using young adult mice minimizes confounding effects of aging, ensuring that observed changes in mitophagy are due to the treatment (saponins from Korean Red Ginseng) rather than developmental changes. Additionally, this age group provides a consistent and reproducible model for metabolic interventions, aligning with previous research that demonstrates robust responses to such treatments [39]. The decision to use only male mice in this study is based on established scientific rationale. Male mice are often preferred in metabolic research due to their consistent metabolic responses and lack of hormonal fluctuations that can affect outcomes. Female mice’s estrous cycles introduce variability, complicating the isolation of intervention effects. Additionally, male mice exhibit higher susceptibility to diet-induced insulin resistance and metabolic syndrome, making them suitable models for these conditions. Using male mice minimized variability, enhancing the reliability of our findings on the saponin treatment’s effects [40,41,42].

Mitochondrial health, crucially linked to oxidative stress regulation [33], plays a pivotal role in the development of insulin resistance and metabolic disorders [43]. Our research underscores RG saponin’s effectiveness in reducing mtROS and total ROS production in hepatocytes, highlighting its protective role against oxidative stress. This decrease in oxidative stress markers, notably the downregulation of P-JN K expression, suggests RG saponin’s potential as a safeguard against oxidative damage. Since P-JNK is associated with the inhibition of insulin signaling [44], its reduction by RG saponin may contribute to the improvement of insulin sensitivity. This aligns with the scientific understanding that unchecked oxidative stress can severely impact metabolic health [45,46], emphasizing the value of antioxidants in counteracting these effects.

Building on this, our study reveals that RG saponin significantly enhances insulin signaling, a key component of metabolic regulation. By upregulating P-IRS and P-AKT, and downregulating gluconeogenic enzymes, RG saponin demonstrates its capacity to improve insulin sensitivity and promote metabolic homeostasis. The modulation of P-JNK, the aforementioned factor known to cause insulin resistance when upregulated, is particularly noteworthy. Its downregulation is likely a contributing factor to the observed enhancement of insulin signaling, reinforcing the connection between reduced oxidative stress and improved insulin signaling function. These findings highlight RG saponin’s multifaceted role in modulating metabolic pathways and its potential as a natural treatment for managing metabolic disorders, indicating its broad-spectrum benefits in improving insulin sensitivity and mitigating the adverse effects of oxidative stress.

However, our study faces limitations, including the inability to conduct in vivo studies specifically addressing insulin resistance, which remains an area for future investigation to fully understand RG saponin’s effects in a physiological context. Additionally, our reliance on the total extract of RG, rather than isolating specific active compounds, poses another limitation, underscoring the need for further research to identify and test individual components for their efficacy. Despite these limitations, our findings affirm the significant potential of RG saponin extract as a natural treatment for enhancing insulin sensitivity and managing metabolic disorders, paving the way for future explorations to harness its full benefits.

## 5. Conclusions

Our study shows that saponins from Korean Red Ginseng improve insulin signaling by enhancing mitophagy, which in turn regulates mitochondrial dynamics and reduces oxidative stress. Through a series of in vitro and in vivo experiments, we observed that treatment with RG saponins led to increased expression of PINK1 and Parkin, critical regulators of mitophagy. This activation of mitophagy was associated with a reduction in both mitochondrial and total reactive oxygen species, alleviating oxidative stress and thereby improving insulin sensitivity. Additionally, we noted significant improvements in insulin signaling pathways, including the upregulation of phosphorylated IRS and AKT and the downregulation of gluconeogenic enzymes. These findings underscore the potential of RG saponins to address insulin resistance and its related metabolic disorders. By identifying this specific molecular effect, our research highlights the promising role of natural compounds in modulating metabolic pathways, suggesting that RG saponins could be a valuable option for improving metabolic health.

## Data Availability

The original contributions presented in the study are included in the article/supplementary material, further inquiries can be directed to the corresponding author.

## Supplementary Materials

The following supporting information can be downloaded at: https://www.mdpi.com/article/10.3390/foods13132137/s1.
