# Supplementary material for: Korean Red Ginseng Improves Oxidative Stress-Induced Hepatic Insulin Resistance via Enhancing Mitophagy
Source: Foods. 2024 Jul 5;13(13):2137. doi: 10.3390/foods13132137 (PMC11241528; doi:10.3390/foods13132137)
Supplement: Supplementary file 1 [file foods-13-02137-s001.zip › foods-3055614-supplementary.pptx]

## Slide 1
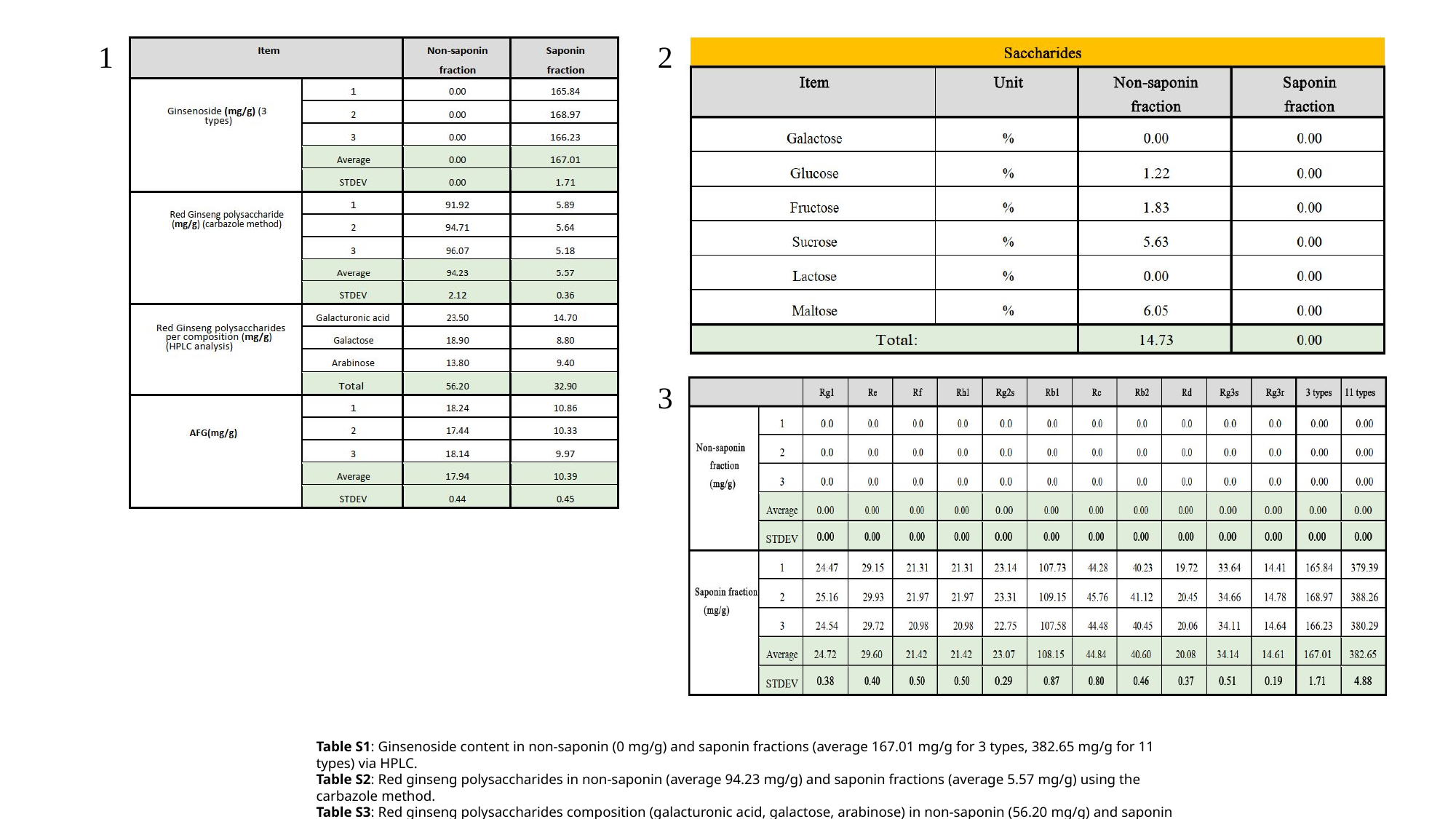

1
2
3
Table S1: Ginsenoside content in non-saponin (0 mg/g) and saponin fractions (average 167.01 mg/g for 3 types, 382.65 mg/g for 11 types) via HPLC.
Table S2: Red ginseng polysaccharides in non-saponin (average 94.23 mg/g) and saponin fractions (average 5.57 mg/g) using the carbazole method.
Table S3: Red ginseng polysaccharides composition (galacturonic acid, galactose, arabinose) in non-saponin (56.20 mg/g) and saponin fractions (32.90 mg/g) via HPLC.
